# Desertification Drives Functional Reassembly of Rhizosphere Fungal Communities from Arbuscular Mycorrhizal Fungi to Dark Septate Endophytes in Temperate Grassland

**DOI:** 10.3390/jof12060440

**Published:** 2026-06-16

**Authors:** Xue Wang, Ruixia Liu, Hui Li, Qingzhi Yao

**Affiliations:** 1 Key Laboratory of Grassland Resources, Ministry of Education, College of Grassland Science, Inner Mongolia Agricultural University, Hohhot 010011, China; wxue4371@gmail.com (X.W.);; 2 Inner Mongolia Shiliuyuan Technology Company Limited, Hohhot 010011, China; 3 Luliang Center for Disease Control and Prevention, Luliang 033000, China

**Keywords:** desertification, rhizosphere fungi, functional reassembly, arbuscular mycorrhizal fungi, dark septate endophytes

## Abstract

Desertification strongly alters soil microbial communities in dryland ecosystems, yet the reassembly of fungal functional groups and their interactions under increasing aridity remain unclear. This study aimed to determine how desertification reshapes arbuscular mycorrhizal fungi (AMF) and endophytic fungal groups in the rhizosphere of *Stipa breviflora* and *Artemisia frigida*, as well as how these shifts are associated with fungal network fragmentation. Rhizosphere soil internal transcribed spacer (ITS) sequencing and AMF-specific amplicon sequencing, combined with root colonization assessment, functional annotation, co-occurrence network analysis, and partial least squares path modeling (PLS-PM), were used to assess shifts in fungal communities along the desertification gradient. Desertification significantly reduced soil multifunctionality and fungal diversity, accompanied by a shift in community composition from environmentally sensitive taxa to stress-tolerant groups. Along the desertification gradient, AMF diversity and colonization decreased, whereas FUNGuild-inferred endophytic fungal abundance and microscopically observed dark septate endophytes (DSEs) colonization increased. FUNGuild-inferred endophytic fungal abundance was negatively correlated with AMF diversity. Co-occurrence networks showed reduced connectivity and increased fragmentation under desertification, especially at the desert steppe and steppe desert stages. PLS-PM analysis revealed that desertification directly increased fungal network fragmentation and indirectly promoted fragmentation through increased FUNGuild-inferred endophytic fungi and reduced AMF diversity, whereas soil multifunctionality mainly reflected environmental deterioration along the gradient. These findings demonstrate the functional reassembly of rhizosphere fungi under desertification and suggest that compensatory shifts among fungal guilds may contribute to ecosystem stability in dryland grasslands.

## 1. Introduction

Soil fungal communities are key components of terrestrial ecosystems, playing fundamental roles in nutrient cycling, organic matter decomposition, and plant health, thereby contributing to ecosystem productivity and stability [1,2]. Based on trophic strategies, fungi are generally classified into symbiotic, saprotrophic, and pathogenic groups, each exhibiting distinct responses to environmental change [3]. Increasing evidence suggests that ecosystem stability is more closely linked to the dynamics of fungal functional groups than to taxonomic diversity alone, supporting the concept of functional redundancy in microbial systems [4]. Therefore, understanding how fungal functional groups respond to environmental stress is essential for predicting ecosystem functioning under global change.

Grasslands in Inner Mongolia represent a typical semi-arid ecosystem and play a crucial role in regional ecological security and sustainable livestock production. However, these ecosystems are increasingly threatened by desertification driven by climate change and human activities [5,6]. Along natural desertification gradients, vegetation structure, soil properties, and microbial communities undergo substantial changes [7,8]. Among dominant plant species, *Stipa breviflora* and *Artemisia frigida* differ markedly in life-history strategies and resource-use patterns, often exhibiting asymmetric responses to environmental stress, which are considered key drivers of community succession in desert steppes [9,10]. Despite this, most studies have treated plant communities as a whole, overlooking species-specific below-ground microbial responses.

Compared with vegetation and soil properties, soil fungal communities are particularly sensitive to desertification processes [11,12]. Previous studies have shown that increasing aridity tends to reduce fungal network complexity and alter community composition, favoring stress-tolerant taxa [13,14]. Arbuscular mycorrhizal fungi (AMF), which enhance plant nutrient acquisition and stress resistance, often decline under grazing and desertification pressures [15,16]. In contrast, certain saprotrophic and endophytic fungi, particularly dark septate endophytes (DSEs), may increase in abundance under dry and nutrient-poor conditions and contribute to plant health by enhancing stress tolerance or suppressing soil-borne pathogens [17,18]. These contrasting responses suggest potential compensatory dynamics among fungal functional groups; however, the extent to which such functional replacement occurs, and how it influences microbial interaction networks, remains poorly understood.

Two major knowledge gaps remain. First, little is known about how rhizosphere fungal communities associated with different functional plant species respond to desertification gradients. Second, the potential compensatory mechanisms among fungal functional groups, particularly under AMF decline, have not been systematically evaluated. To address these gaps, we investigated rhizosphere fungal communities associated with *Stipa breviflora* and *Artemisia frigida* along a natural desertification gradient to determine how desertification alters fungal functional groups and fungal association networks, particularly network connectivity and fragmentation.

## 2. Materials and Methods

### 2.1. Study Area

The study was conducted in Siziwang Banner, Inner Mongolia, China, along a north–south transect (41°42′–42°32′ N, 110°59′–112°28′ E) at elevations ranging from 1144 to 1450 m. Across the sampling transect, the mean annual temperature varies spatially from approximately 1 to 6 °C, and annual precipitation ranges from 110 to 350 mm. Evaporation exceeds precipitation by approximately 8–10 times, reflecting strong aridity.

The landscape is topographically heterogeneous, comprising the northern foothills of the Yinshan Mountains, the Ulanqab Hills, and the Mongolian Plateau from south to north. This environmental heterogeneity supports distinct grassland types distributed along the transect. According to the Chinese grassland classification system (NY/T 2997-2016), the study area can be divided into three vegetation zones: typical steppe (southern region), desert steppe (intermediate zone), and steppe desert (northern region). These zones form a natural desertification gradient, providing an ideal system for investigating ecological responses to increasing aridity.

### 2.2. Sample Collection

**Figure 1:**
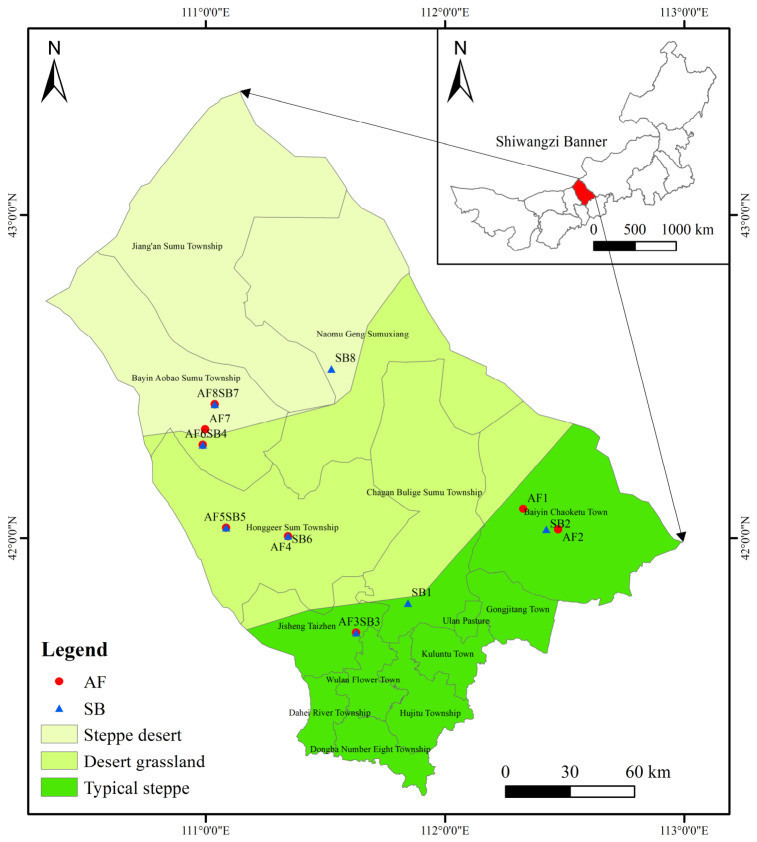
Sampling Site Information Map.

Sampling was conducted in August 2019 during the peak growing season. Two dominant plant species, *Stipa breviflora* and *Artemisia frigida*, were selected across three desertification stages: typical steppe (TS), desert steppe (DS), and steppe desert (SD). A total of eight sampling sites were established (TS: 3 sites; DS: 3 sites; SD: 2 sites). At each site, three 2 m × 2 m plots were randomly established as independent biological replicates, with a minimum distance of 100 m between plots (Figure 1).

Within each plot, plant roots and rhizosphere soils were collected using a five-point sampling method. Roots were gently shaken to remove loosely attached soil, retaining rhizosphere soil as much as possible. Soil samples were sieved through a 2 mm mesh to remove stones and plant debris, and subsamples from each plot were homogenized to form one composite sample, resulting in a total of 48 soil samples.

Each composite sample was divided into three subsamples: one stored at −80 °C for the DNA extraction and high-throughput sequencing of fungal and arbuscular mycorrhizal fungal (AMF) communities; one stored at 4 °C for soil enzyme activity analysis; one air-dried for soil physicochemical measurements. Root samples were carefully washed and preserved separately for subsequent assessment of fungal colonization.

### 2.3. Soil Properties and Soil Multifunctionality

Soil physicochemical properties were determined according to standard soil agrochemical analysis methods and corresponding Chinese standard protocols [19], as summarized in Table 1. Soil pH was measured in a soil–water suspension at a ratio of 1:2.5 (*w*/*v*) using a pH meter (PHS-3C, Shanghai INESA Scientific Instrument Co., Ltd., Shanghai, China). Soil organic matter (SOM) was determined using the external heating potassium dichromate oxidation method according to NY/T 1211.6-2006. Briefly, soil samples were oxidized with potassium dichromate under acidic heating conditions, and the remaining dichromate was quantified by titration.

Alkali-hydrolyzable nitrogen (AN) was determined using the alkali diffusion method according to LY/T 1228-2015. Soil samples were treated with alkaline solution to release ammonium nitrogen, which was absorbed by boric acid after diffusion under constant-temperature incubation using a constant-temperature incubator (LRH-70F, Shanghai Yiheng Scientific Instrument Co., Ltd., Shanghai, China), followed by titration. Available phosphorus (AP) was extracted with sodium bicarbonate and determined using the molybdenum antimony colorimetric method according to NY/T 1121.25-2012. The absorbance of the phosphomolybdenum blue complex was measured using a microplate reader (Multiskan FC, Thermo Fisher Scientific, Waltham, MA, USA).

Total nitrogen (TN) was determined using the Kjeldahl method according to NY/T 1121.24-2012. Soil samples were digested with concentrated sulfuric acid, and nitrogen was quantified using an automatic Kjeldahl analyzer (Kjeltec 8100, FOSS, Hillerød, Denmark). Total phosphorus (TP) was determined by the alkali fusion–molybdenum antimony colorimetric method according to NY/T 88-1988. Briefly, soil samples were fused with alkali, and phosphate released after digestion was measured colorimetrically using a microplate reader (Multiskan FC, Thermo Fisher Scientific, USA). Total potassium (TK) was determined using the sodium hydroxide fusion method according to NY/T 87-1988, and potassium concentration was measured using a multi-element flame photometer (FP6450, Shanghai Yoke Instrument Co., Ltd., Shanghai, China).

Soil enzyme activities, including urease (URE), sucrase (SUC), acid phosphatase (ACP), and alkaline phosphatase (ALP), were determined by spectrophotometry using commercial assay kits according to the manufacturer’s instructions (Solarbio, Beijing, China). Absorbance was measured using a microplate reader (Multiskan FC, Thermo Fisher Scientific, USA).

To comprehensively assess soil carbon, nitrogen, and phosphorus cycling, as well as nutrient retention capacity, nine commonly used indicators were selected to calculate soil multifunctionality (SMF): AN, AP, SOM, TN, TP, URE, SUC, ACP, and ALP. These variables are widely applied in soil multifunctionality studies and represent key determinants of ecosystem functioning in dryland and grassland systems [20]. SMF was calculated using the averaging approach based on standardized variables [21]. Specifically, each variable was transformed into a Z-score using the following equation:

Z-score = (X − Xmean)/XSD

The SMF index was then obtained by averaging the standardized values of all selected variables.

### 2.4. Root Colonization Assessment

Root colonization by arbuscular mycorrhizal fungi (AMF) and dark septate endophytes (DSEs) was determined using a modified trypan blue staining method based on the work of Phillips and Hayman (1970) [22] Root samples preserved in FAA solution were thoroughly washed and cut into approximately 1 cm segments. Root segments were cleared in 10% KOH at 90 °C to remove cytoplasmic contents and facilitate fungal staining. Depending on root characteristics, clearing time ranged from 20 to 40 min. Pigmented roots were subsequently bleached in alkaline hydrogen peroxide solution and rinsed thoroughly with water. The root segments were then acidified in 5% lactic acid for 5 min and stained with 0.05% trypan blue solution prepared in lactic acid/glycerol/distilled water (1:1:1, *v*/*v*/*v*). Following staining, roots were destained in lactoglycerol solution and mounted on microscope slides using polyvinyl alcohol–lactic acid–glycerol (PVLG).

For each sample, 30 randomly selected root fragments were distributed across three microscope slides. Colonization was quantified using the line-intersect method described by Giovannetti and Mosse (1980) [23]. Microscopic observations were performed under a light microscope, and at least 150 intersections were examined per sample. The presence of AMF hyphae, vesicles, as well as DSE melanized hyphae and microsclerotia, was recorded separately. Representative fungal structures were photographed for documentation.

To minimize observer bias, all samples were randomly coded prior to microscopic examination, and colonization scoring was performed blind to treatment identity.

Colonization rates of AMF hyphae, vesicles, arbuscules, DSE hyphae, and microsclerotia were calculated as the percentage of intersections containing the corresponding fungal structures relative to the total number of intersections observed. Total fungal colonization was calculated as

Total colonization (%) = (Total intersections − Non-colonized intersections)/Total intersections × 100

### 2.5. DNA Extraction, Amplification, and Sequencing

The diversity and community structure of rhizosphere fungi and arbuscular mycorrhizal fungi (AMF) were analyzed using high-throughput amplicon sequencing. Total genomic DNA was extracted from rhizosphere soil samples using the cetyltrimethylammonium bromide (CTAB) method [24]. DNA concentration and purity were determined using a NanoDrop spectrophotometer (Thermo Fisher Scientific, Waltham, MA, USA), and DNA integrity was checked by 1.2% agarose gel electrophoresis.

For the general fungal community, the fungal ITS1 region was amplified using the primer pair ITS5 (5′-GGAAGTAAAAGTCGTAACAAGG-3′) and ITS2 (5′-GCTGCGTTCTTCATCGATGC-3′). For the AMF community, AMF-specific amplicons were amplified using the primer pair AMV4.5NF (5′-AAGCTCGTAGTTGAATTTCG-3′) and AMDGR (5′-CCCAACTATCCCTATTAATCAT-3′) [25]. Sample-specific barcode sequences were incorporated into the primers to distinguish different samples.

PCR amplification was performed using Pfu high-fidelity DNA polymerase (TransGen Biotech, Beijing, China). The number of PCR cycles was minimized while ensuring sufficient product yield, and all samples within the same batch were amplified under consistent PCR conditions. Negative controls were included during PCR amplification to monitor potential contamination from reagents or the laboratory environment.

PCR products were purified using VAHTS DNA Clean Beads (Vazyme, Nanjing, China). Purified amplicons were quantified using the Quant-iT PicoGreen dsDNA Assay Kit (Invitrogen, Carlsbad, CA, USA) on a BioTek FLx800 microplate reader (BioTek Instruments, San Diego, CA, USA). Based on the fluorescence quantification results, amplicons from different samples were pooled in equimolar concentrations according to the required sequencing depth.

Sequencing libraries were prepared using the TruSeq Nano DNA LT Library Prep Kit (Illumina, San Diego, CA, USA). Briefly, purified amplicons were subjected to end repair, A-tailing, adapter ligation, PCR enrichment, and purification using AMPure XP beads (Beckman Coulter, Brea, CA, USA). Library quality was evaluated using an Agilent Bioanalyzer with the Agilent High Sensitivity DNA Kit (Agilent Technologies, Santa Clara, CA, USA). Library concentrations were further quantified using the Quant-iT PicoGreen dsDNA Assay Kit on a Promega QuantiFluor fluorescence quantification system (Promega, Fitchburg, WI, USA). Only qualified libraries with a single peak, no detectable adapter contamination, and concentrations above 2 nM were used for sequencing.

Qualified libraries were pooled in appropriate proportions, denatured with NaOH into single-stranded DNA, and sequenced on the Illumina MiSeq platform using paired-end sequencing. Sequencing was performed by Personal Biotechnology Co., Ltd. (Shanghai, China). Raw sequence data were generated in FASTQ format and deposited in the NCBI Sequence Read Archive (SRA) under BioProject accession number PRJNA1455379.

### 2.6. Bioinformatics and Community Analyses

Microbiome bioinformatics analyses were performed using QIIME2 v2019.4 [26]. The fungal ITS and AMF amplicon datasets were processed separately.

For the fungal ITS dataset, sequences were quality-filtered, denoised, merged, and chimera-checked using the DADA2 plugin [27]. Singleton sequence features were removed to minimize potential sequencing artifacts. Representative fungal ITS sequences were taxonomically assigned using the classify-sklearn naïve Bayes classifier in the q2-feature-classifier plugin [28] against the UNITE database version 8 [29]. Taxa assigned to non-fungal lineages, including plants and other non-target organisms, were removed prior to downstream analyses.

For the AMF dataset, sequence processing was performed using the vsearch pipeline [30]. Briefly, paired-end reads were merged, quality-filtered, dereplicated, clustered, and chimera-checked. Representative AMF sequences were taxonomically classified against the curated MaarjAM database specifically developed for arbuscular mycorrhizal fungi [31], Non-AMF sequences and unclassified non-target sequences were removed before downstream analyses.

To ensure comparability among samples, fungal ITS and AMF feature tables were rarefied before downstream analyses. Alpha diversity indices, including Chao1, Observed species, Shannon, Simpson, and Good’s coverage, were calculated based on the rarefied feature tables. Beta diversity was assessed using Bray–Curtis distance matrices and visualized by non-metric multidimensional scaling (NMDS) Community dissimilarities among groups were evaluated using analysis of similarities (ANOSIM) and permutational multivariate analysis of variance (PERMANOVA, ADONIS).

Fungal functional guilds were assigned using FUNGuild based on the fungal ITS taxonomic table [32]. To reduce uncertainty associated with functional prediction, only taxa assigned with “highly probable” and “probable” confidence rankings were retained for downstream analyses, whereas “possible” assignments and unassigned taxa were excluded. In this study, the term “FUNGuild-inferred endophytic fungi” refers to broad endophytic fungal guilds inferred from ITS amplicon data. These FUNGuild-inferred endophytic fungi were not directly equated with dark septate endophytes (DSEs), which were independently assessed based on root staining and microscopic observation.

Random forest analysis was performed using the q2-sample-classifier plugin in QIIME2 v2019.4 based on genus-level abundance tables generated from taxonomic assignments [33]. The “classify_samples_ncv” function was used with the default random forest classifier and nested stratified cross-validation. Important fungal and AMF taxa were identified according to their contribution to classification accuracy.

Co-occurrence networks were constructed to examine fungal interaction patterns across desertification stages. Sequence features detected in more than 20% of samples and with relative abundance greater than 0.01% were retained for network analysis. Pairwise Spearman correlations among sequence features were calculated, and significant correlations after Benjamini–Hochberg correction [34] were used to construct weighted undirected networks. Network topological properties, including node number, edge number, average degree, average shortest path length, network diameter, network density, clustering coefficient, modularity, network complexity indices, and number of connected components, were calculated to characterize fungal network structure [35].

To reduce the uncertainty associated with small sample sizes in plant species × desertification stage-specific networks, co-occurrence networks were constructed at the desertification-stage level by pooling rhizosphere samples from both plant species within each stage. This pooled design increased the sample size for each network and allowed more robust comparison of network topology across desertification stages.

### 2.7. Statistical Analyses

All statistical analyses were conducted using SPSS v26.0 and R v4.5.0, and data visualization was performed using the ggplot2 package in R and Origin 2021. Differences in soil properties, soil multifunctionality, root colonization, microbial diversity indices, fungal functional guilds, and network topological properties among desertification stages (TS, DS, and SD) were tested using one-way analysis of variance (ANOVA), followed by LSD or Duncan’s multiple comparison tests where appropriate, with significant differences indicated by different letters. Two-way ANOVA was used to assess the effects of desertification stage, plant species, and their interaction on soil properties and fungal functional guilds.

To evaluate the effects of desertification on fungal and AMF alpha diversity, linear mixed-effects models were fitted using the lme4 package, with desertification stage set as a fixed factor and plant species as a random factor under a Gaussian distribution [36]. Spearman correlation analysis was used to assess associations among fungal functional guilds, AMF diversity, DSE colonization characteristics, soil multifunctionality, and network topological properties [37]. Significant relationships were further evaluated using ordinary least squares (OLS) regression where appropriate, and the coefficient of determination (R^2^) and regression equations are presented in the corresponding figures.

Partial least squares path modeling (PLS-PM) was performed to evaluate the direct and indirect effects of desertification on fungal functional groups, soil multifunctionality, AMF diversity, and fungal network fragmentation [38]. Statistical significance was defined as *p* < 0.05. In the figures, significance levels were indicated as follows: * *p* < 0.05, ** *p* < 0.01, and *** *p* < 0.001.

## 3. Results

### 3.1. Effects of Desertification on Soil Properties and Soil Multifunctionality

**Figure 2:**
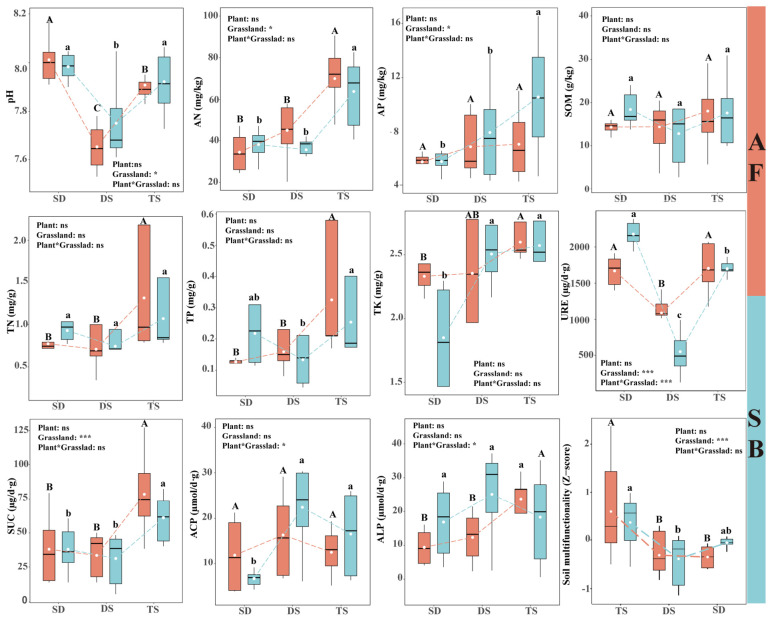
Changes in rhizosphere soil physicochemical properties and soil multifunctionality (SMF) of *Stipa breviflora* (SB) and *Artemisia frigida* (AF) across desertification stages. Boxplots show the distribution of values, with the central line indicating the median and points representing the mean. Different lowercase letters indicate significant differences among desertification stages within SB, and different uppercase letters indicate significant differences within AF (*p* < 0.05). The results of two-way ANOVA show the effects of desertification (Grassland), plant species (Plant), and their interaction (Grassland × Plant): ns, not significant; * *p* < 0.05; *** *p* < 0.001.

Desertification and plant species significantly influenced soil pH, available nutrients, and enzyme activities, which exhibited a unimodal pattern with the lowest values observed at the desert steppe (DS) stage (Figure 2). Compared with the typical steppe (TS), soil pH, urease (URE), sucrase (SUC), available nitrogen (AN), and available phosphorus (AP) significantly decreased at the DS stage, with reductions ranging from 35.94% to 67.52%. In contrast, alkaline phosphatase (ALP) and acid phosphatase (ACP) activities significantly increased, with increments of 7.88% to 30.07%.

Correspondingly, soil multifunctionality (SMF) showed a sharp decline at the DS stage. The maximum reductions in SMF were 1.59-fold and 2.05-fold in *Artemisia frigida* (AF) and *Stipa breviflora* (SB), respectively, indicating a substantial deterioration of below-ground ecosystem functioning under desertification.

### 3.2. Changes in Soil Fungal Diversity and Community Composition Along the Desertification Gradient

**Figure 3:**
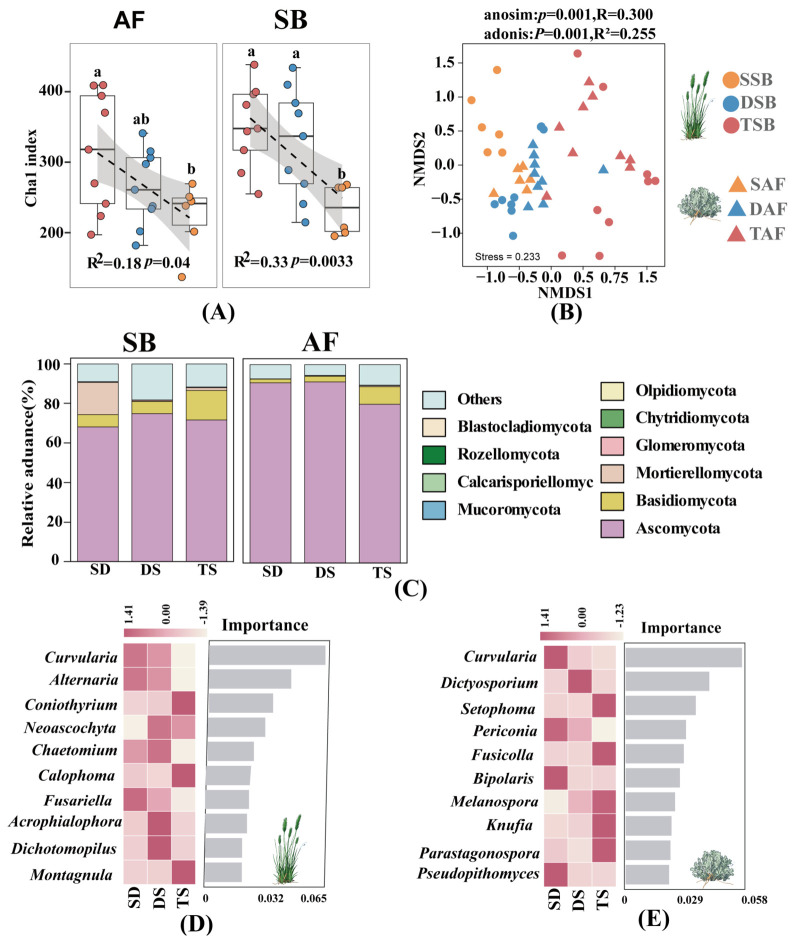
Effects of desertification on rhizosphere fungal diversity and community structure in Stipa breviflora (SB) and Artemisia frigida (AF). (**A**) Alpha diversity of fungal communities. Regression lines and equations are based on linear models, and significance was evaluated using linear mixed-effects models (LMMs) with desertification as a fixed factor and plant species as a random factor. Different lowercase letters indicate significant differences among groups (*p* < 0.05). The color legend is the same as in panel (**B**). (**B**) Non-metric multidimensional scaling (NMDS) based on Bray–Curtis distance showing beta diversity, with group differences tested by ANOSIM and ADONIS. (**C**) Relative abundance of dominant fungal phyla across desertification stages. (**D**) Key fungal genera identified by random forest analysis at the genus level for AF rhizospheres. (**E**) Key fungal genera identified by random forest analysis at the genus level for SB rhizospheres.

Fungal alpha diversity significantly decreased with increasing desertification (AF: R^2^ = 0.18, *p* = 0.04; SB: R^2^ = 0.33, *p* = 0.0033; Figure 3A). Compared with the typical steppe (TS), the Chao1 index at the steppe desert (SD) stage decreased by 33.70% and 28.97% in *Artemisia frigida* (AF) and *Stipa breviflora* (SB), respectively.

Non-metric multidimensional scaling (NMDS), together with ANOSIM and ADONIS tests, revealed significant differences in fungal community composition among desertification stages (Figure 3B). At the phylum level, Ascomycota (relative abundance: 0.68–0.91) and Basidiomycota (0.02–0.15) consistently dominated across all stages (Figure 3C). Environmental driver analysis showed that fungal diversity in SB was mainly associated with total potassium (TK), acid phosphatase (ACP) and total phosphorus (TP), whereas that in AF was primarily influenced by alkaline phosphatase (ALP) (Figures S1 and S2).

Random forest analysis further indicated clear shifts in community composition along the desertification gradient. In AF rhizospheres, *Curvularia*, *Alternaria*, and *Chaetomium* were enriched at the SD stage, whereas TS-associated genera such as *Coniothyrium*, *Neoascothyta*, and *Calophoma* were no longer detected (Figure 3D). Similarly, in SB rhizospheres, *Curvularia*, *Periconia*, *Bipolaris*, and *Pseudopithomyces* were enriched at the SD stage, whereas TS-associated genera including *Setophoma*, *Fusicolla*, and *Melanospora* disappeared (Figure 3E).

### 3.3. Changes in AMF and Fungal Functional Guilds Along the Desertification Gradient

**Figure 4:**
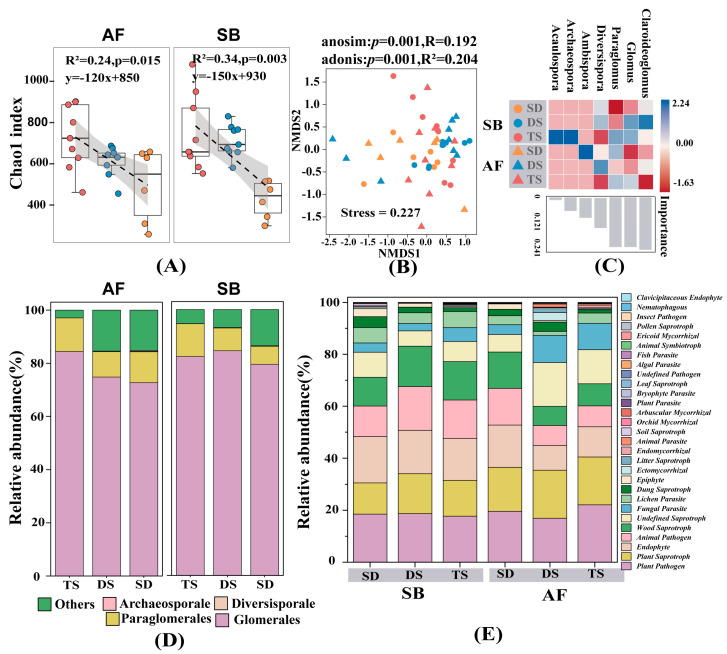
Effects of desertification on arbuscular mycorrhizal fungal (AMF) communities in the rhizosphere of *Stipa breviflora* (SB) and *Artemisia frigida* (AF). (**A**) Alpha diversity of AMF communities. Regression lines and equations are based on linear models, and significance was evaluated using linear mixed-effects models (LMMs) with desertification as a fixed factor and plant species as a random factor. The circle colors are the same as those in panel (**C**). (**B**) Non-metric multidimensional scaling (NMDS) based on Bray–Curtis distance showing AMF beta diversity, with group differences tested by ANOSIM and ADONIS. (**C**) Relative abundance of dominant AMF orders across desertification stages. (**D**) Key AMF genera identified by random forest analysis at the genus level for SB and AF rhizospheres. (**E**) Functional guild composition based on FUNGuild, showing changes in major functional groups along the desertification gradient.

The alpha diversity of arbuscular mycorrhizal fungi (AMF) significantly decreased along the desertification gradient (AF: R^2^ = 0.24, *p* = 0.015; SB: R^2^ = 0.33, *p* = 0.003; Figure 4A). Compared with the typical steppe (TS), the Chao1 index of AMF decreased by 42.67% and 27.21% in the steppe desert (SD) stage for *Artemisia frigida* (AF) and *Stipa breviflora* (SB), respectively.

Non-metric multidimensional scaling (NMDS), combined with ANOSIM and ADONIS tests, revealed significant differences in AMF community composition among desertification stages (Figure 4B). At the taxonomic level, Glomerales consistently dominated the AMF communities, with relative abundances ranging from 0.73 to 0.85 (Figure 4C). Random forest analysis further showed that AMF biomarker taxa were largely absent at the SD stage (Figure 4D).

Functional annotation based on the FUNGuild database identified 29 fungal functional guilds (Figure 4E). Desertification significantly altered the relative abundances of several functional groups, including plant saprotrophs, endophytes, animal pathogens, wood saprotrophs, undefined saprotrophs, fungal parasites, and lichen parasites, whereas plant species had no significant effect (Table S1).

**Figure 5:**
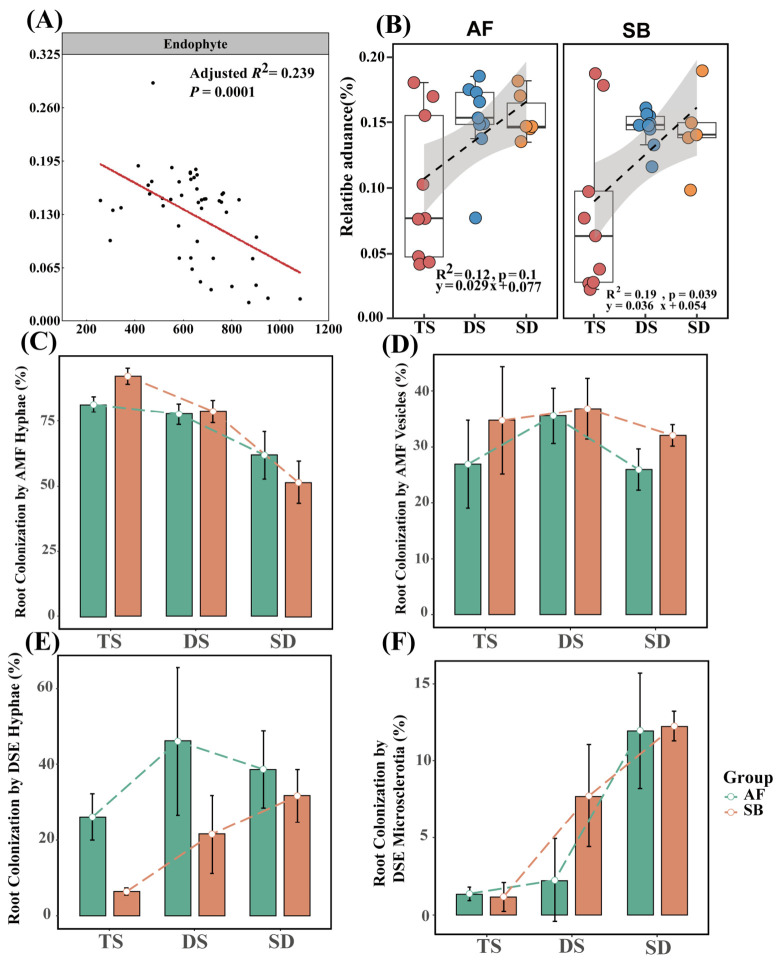
Relationships between arbuscular mycorrhizal fungi (AMF) and endophytic fungi (dark septate endophytes, DSE) and their root colonization patterns under desertification. (**A**) Linear regression between AMF diversity and endophytic fungal abundance. (**B**) Changes in endophytic fungal abundance along the desertification gradient. (**C**–**F**) Root colonization characteristics measured by staining and microscopy, including (**C**) AMF vesicle colonization, (**D**) AMF hyphal colonization, (**E**) DSE hyphal colonization, and (**F**) DSE microsclerotia colonization.

Correlation analysis revealed significant negative relationships between AMF diversity and several fungal functional groups, including endophytes, wood saprotrophs, animal pathogens, lichen parasites, etc. (Figure S3). Among these, endophytes showed the strongest negative correlation with AMF diversity (R^2^ = 0.239, *p* = 0.0001; Figure 5A). In addition, the relative abundance of endophytic fungi increased along the desertification gradient, with increases of 110.45% and 55.48% at the desert steppe (DS) stage in SB and AF rhizospheres, respectively (Figure 5B).

**Figure 6:**
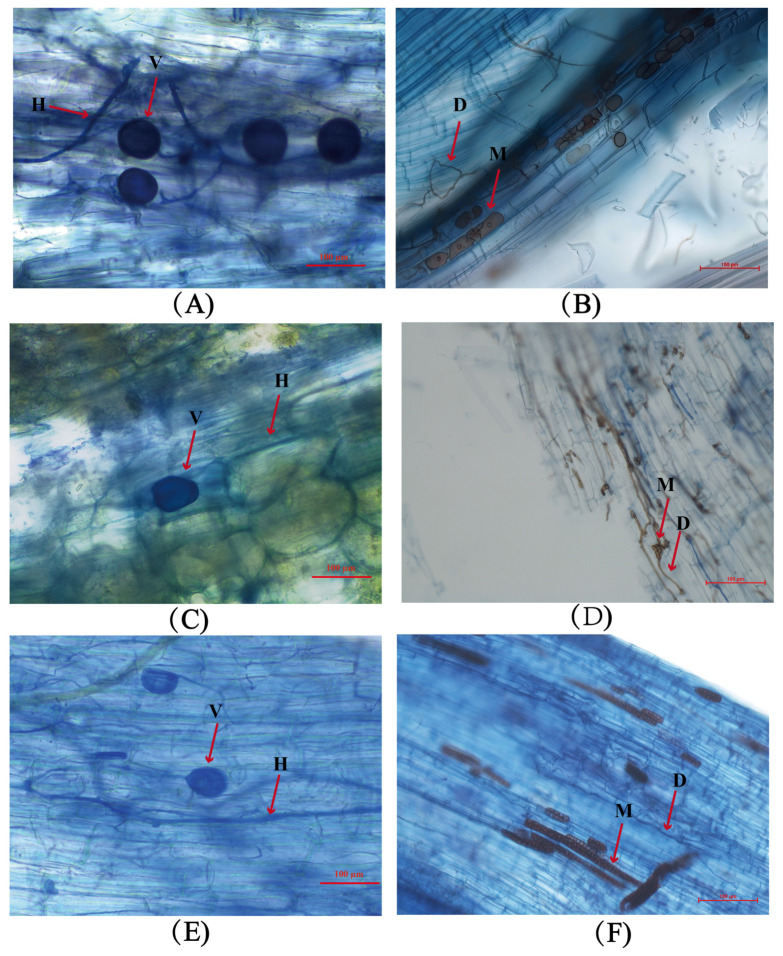
Root colonization characteristics of arbuscular mycorrhizal fungi (AMF) and dark septate endophytes (DSEs) under different degrees of desertification: (**A**) AMF colonization in TS; (**B**) DSE colonization in TS; (**C**) AMF colonization in DS; (**D**) DSE colonization in DS; (**E**) AMF colonization in SD; (**F**) DSE colonization in SD. H, AMF hyphae; V, AMF vesicles; D, DSE hyphae; M, microsclerotia. TS, typical steppe; DS, desert steppe; SD, steppe desert.

Root colonization analysis further supported these patterns. With increasing desertification, AMF vesicle and hyphal colonization rates decreased (Figure 5C,D and Figure 6), whereas the colonization rates of dark septate endophytes (DSEs), including hyphae and microsclerotia, increased significantly (Figure 5E,F and Figure 6).

### 3.4. Changes in Fungal Co-Occurrence Networks and Their Potential Drivers

**Figure 7:**
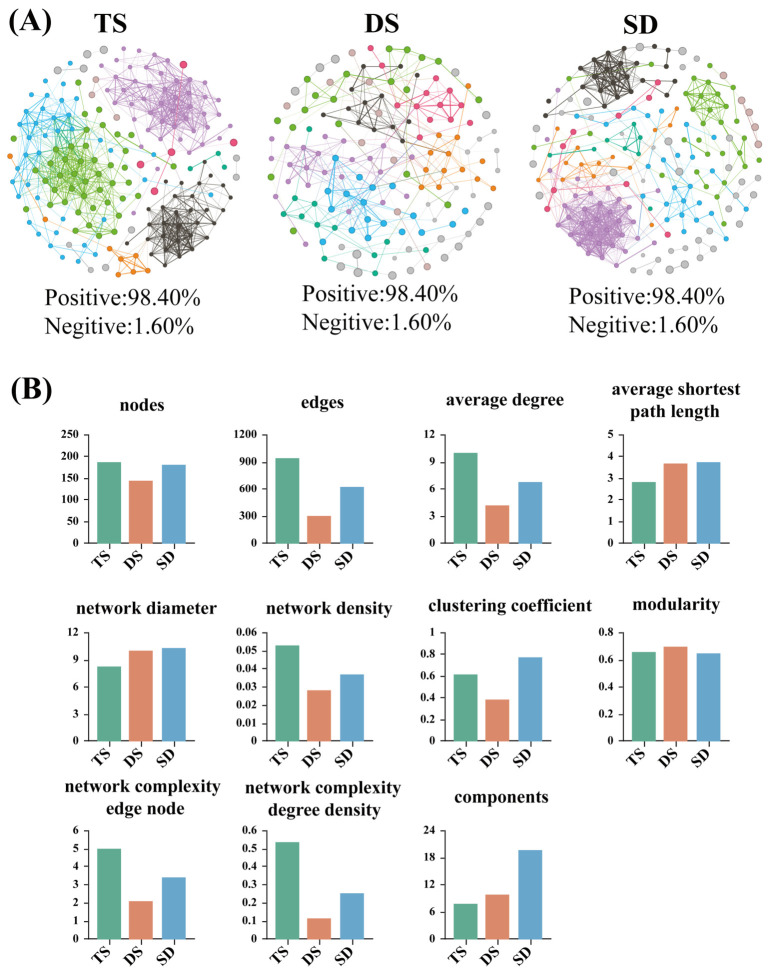
Effects of desertification on rhizosphere soil fungal co-occurrence networks. (**A**) Visualization of stage-level fungal co-occurrence networks constructed by pooling rhizosphere samples from both plant species within each desertification stage. Different node colors indicate different network modules. The proportions of positive and negative correlations are shown below each network. (**B**) Changes in network topological properties across desertification stages, including node number, edge number, average degree, average shortest path length, network diameter, network density, clustering coefficient, modularity, network complexity indices, and number of connected components. TS, typical steppe; DS, desert steppe; SD, steppe desert.

Desertification markedly altered the co-occurrence patterns of rhizosphere fungal communities (Figure 7). With increasing desertification, network topology exhibited a threshold-like shift at the desert steppe (DS) stage, characterized by a sharp reduction in network connectivity. The pooled network analysis showed that network connectivity decreased markedly at the DS stage, followed by partial recovery at the steppe desert (SD) stage. Compared with TS, the DS network showed decreases of 22.11%, 67.19%, 57.88%, and 45.84% in node number, edge number, average degree, and network density, respectively. At the SD stage, these connectivity-related indices partially recovered compared with DS; however, edge number, average degree, and network density remained 33.33%, 31.53%, and 29.67% lower than those in TS, respectively.

Meanwhile, average shortest path length, network diameter, and the number of connected components increased with increasing desertification, indicating progressive network fragmentation. Overall, desertification simplified fungal networks by reducing connectivity and further promoted network fragmentation, with the strongest loss of connectivity occurring at the DS stage and the highest fragmentation occurring at the SD stage.

Correlation analyses indicated that both FUNGuild-inferred endophytic fungi and AMF were significantly associated with network fragmentation-related topological properties, particularly average shortest path length, network diameter, and the number of connected components (Figure S4). Higher values of these indices indicate longer connection paths, greater network separation, and stronger fragmentation of fungal association networks, whereas no significant relationships were detected between soil physicochemical properties and these network parameters (Figure S5).

**Figure 8:**
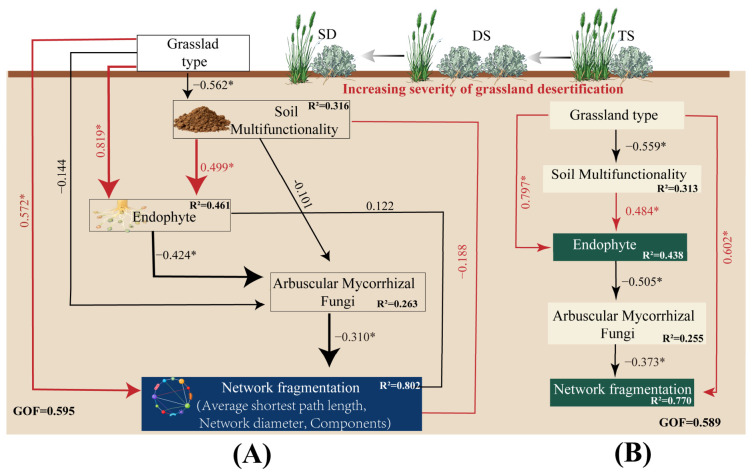
Partial least squares path modeling (PLS-PM) illustrating the relationships among desertification, fungal functional groups, soil multifunctionality (SMF), and fungal network fragmentation. Network topological properties closely associated with fungal functional groups, including average shortest path length, network diameter, and the number of connected components, were integrated into a composite index of network fragmentation. (**A**) Initial conceptual model showing the potential relationships among desertification, SMF, fungal functional groups, and network fragmentation. (**B**) Final model after removal of non-significant paths.* *p* < 0.05.

Partial least squares path modeling (PLS-PM) further revealed the potential drivers of fungal network fragmentation (Figure 8). In the initial model, desertification, SMF, and AMF diversity exerted direct effects on network fragmentation, whereas endophytic fungal abundance mainly acted indirectly through its negative association with AMF diversity (Figure 8A). After removing non-significant paths, the simplified model showed that desertification directly increased endophytic fungal abundance and network fragmentation, while also indirectly promoting network fragmentation through the pathway of increased endophytic fungi and reduced AMF diversity (Figure 8B).

## 4. Discussion

### 4.1. Environmental Filtering Drives Fungal Community Reassembly Under Desertification

Our results demonstrate that soil multifunctionality sharply declined at the desert steppe stage, indicating a critical threshold in below-ground ecosystem functioning. This nonlinear response contrasts with the linear decline commonly reported in small-scale studies [39] or the intermediate disturbance hypothesis [40], and instead supports a threshold response along aridity gradients [41]. As a transitional and ecologically fragile zone [42], the desert steppe is highly susceptible to the combined effects of increased aridity and grazing pressure, which accelerate soil erosion and nutrient loss [43] and ultimately enhance environmental heterogeneity across desertification stages [44].

Fungal alpha diversity declined significantly with increasing desertification, consistent with patterns observed in degraded grasslands under long-term grazing [45]. This finding supports the environmental filtering hypothesis [46], whereby extreme conditions selectively eliminate sensitive taxa. Mechanistically, this process is likely driven by reduced nutrient availability, which limits microbial metabolic activity [47], and by water limitation, which constrains ecological niche space [48].

Changes in fungal indicator taxa further highlight adaptive community reassembly. With increasing desertification, fungal communities shifted from environmentally sensitive saprotrophic taxa [49] (e.g., *Setophoma* and *Fusicolla*) toward stress-tolerant taxa, including genera previously reported to include DSE representatives [50] (e.g., *Curvularia*). This transition is consistent with a shift from resource-acquisitive to stress-tolerant strategies, as predicted by CSR theory. DSE fungi are often characterized by melanized hyphae and microsclerotia, traits that enhance resistance to UV radiation, oxidative stress, and desiccation [51]. In this study, the increase in DSE colonization was further supported by root staining and microscopic observation.

### 4.2. Divergent Responses of AMF, FUNGuild-Inferred Endophytic Fungi, and DSE Colonization

We observed a consistent decline in arbuscular mycorrhizal fungi (AMF) diversity, accompanied by an increase in FUNGuild-inferred endophytic fungi, suggesting potential niche differentiation in root-associated fungal communities under desertification. Although direct evidence remains limited, AMF and endophytic fungi may compete for root colonization sites and host-derived resources under drought conditions [52]. It should be noted that FUNGuild-inferred endophytic fungi represent broad functional guilds predicted from ITS amplicon data and should not be directly equated with dark septate endophytes (DSEs); DSE responses in this study were supported by root staining and microscopic observation.

As obligate symbionts, AMF depend on host-derived carbon, particularly lipid transfer from plant photosynthesis [53,54]. Desertification-induced drought may reduce plant carbon assimilation and alter host carbon allocation, thereby weakening AMF symbiosis. AMF are also widely recognized for their roles in enhancing plant nutrient acquisition, particularly phosphorus uptake, and improving plant adaptation to environmental stress. Therefore, the observed decline in AMF diversity and colonization may reflect the sensitivity of mycorrhizal associations to increasing aridity and resource limitation.

In contrast, many endophytic fungi are facultative root-associated fungi with lower dependence on host carbon and broader environmental tolerance [55]. The increase in FUNGuild-inferred endophytic fungi under desertification may therefore reflect the enrichment of fungal guilds better adapted to stressful and nutrient-limited conditions. Phosphatase activity emerged as an important factor associated with fungal community shifts, potentially reflecting differences in phosphorus acquisition strategies between AMF and DSE. While AMF primarily contribute to inorganic phosphorus acquisition, DSE have been reported to participate in organic phosphorus mineralization [56]. Under nutrient-limited conditions, this functional divergence may contribute to a shift from mycorrhizal-dominated associations toward endophyte-associated stress-tolerance strategies.

Microscopic observations further supported this shift in root colonization patterns. In less degraded grasslands, root colonization was dominated by AMF hyphae, which facilitate nutrient acquisition under relatively favorable conditions [57]. With increasing desertification, DSE colonization, particularly microsclerotia formation, increased markedly, whereas AMF colonization declined. Similar patterns have been reported in desert plants such as *Populus euphratica* and *Haloxylon ammodendron* [18]. DSE microsclerotia function as survival structures that store carbon reserves and enhance stress tolerance through melanin production and reactive oxygen species (ROS) regulation [58]. Together, the parallel increase in FUNGuild-inferred endophytic fungi and microscopically observed DSE colonization suggests a coordinated shift in root-associated fungal strategies from nutrient acquisition toward stress tolerance under desertification.

### 4.3. Functional Guild Shifts Are Associated with Network Fragmentation

Desertification significantly altered fungal co-occurrence networks in rhizosphere soils. Increasing desertification reduced fungal network connectivity, as indicated by decreases in edge number, average degree, and network density, consistent with observations in alpine grasslands on the Qinghai–Tibet Plateau [59] and semi-arid agro-pastoral ecotones in Inner Mongolia [60]. Meanwhile, average shortest path length, network diameter, and the number of connected components increased along the desertification gradient, indicating enhanced network fragmentation. This fragmentation may reflect the disruption of fungal associations under resource limitation, leading to weaker connectivity among taxa and greater separation among network components, which is consistent with recent evidence showing that intensified aridity reduces soil microbial network complexity and undermines the role of soil biodiversity in supporting ecosystem stability [61]. Overall, these results suggest that desertification not only simplified fungal association networks but also promoted their fragmentation and reduced network integration.

Importantly, network restructuring was primarily associated with fungal functional groups rather than soil physicochemical properties. Although abiotic factors are often considered key drivers in semi-arid ecosystems [62], our results suggest a decoupling between environmental variables and network topology across the full desertification gradient. PLS-PM analysis further indicated that desertification promoted network fragmentation both directly and indirectly through changes in fungal functional groups, particularly the increase in FUNGuild-inferred endophytic fungi and the decline in AMF diversity. This is consistent with previous studies showing that fungal diversity can influence microbial community structure [63] and that mycorrhizal networks play a central role in ecosystem resistance [64].

It should be noted that co-occurrence networks based on amplicon sequencing data and correlation analysis do not necessarily represent direct ecological interactions. The edges in these networks indicate significant statistical associations rather than confirmed biological interactions. Therefore, the observed reduction in network connectivity and increase in fragmentation should be interpreted as changes in potential fungal association patterns under desertification.

Overall, the contrasting responses of AMF and FUNGuild-inferred endophytic fungi, together with increased DSE colonization observed microscopically, suggest not only a shift in fungal functional composition but also a reorganization of below-ground fungal association networks, resulting in less connected and more fragmented fungal networks under desertification.

## 5. Conclusions

Desertification significantly reduced soil multifunctionality and reshaped rhizosphere fungal communities in temperate grasslands of Inner Mongolia. Along the desertification gradient, fungal communities shifted from environmentally sensitive taxa toward stress-tolerant groups, accompanied by a decline in AMF diversity and colonization, an increase in FUNGuild-inferred endophytic fungi, and enhanced DSE colonization observed by microscopy. These functional guild shifts were associated with reduced network connectivity and increased network fragmentation. PLS-PM further suggested that desertification promoted fungal network fragmentation both directly and indirectly through increased FUNGuild-inferred endophytic fungi and reduced AMF diversity. Overall, our findings indicate that the coordinated shift among AMF decline, increased FUNGuild-inferred endophytic fungi, and enhanced DSE colonization is a key feature of rhizosphere fungal reassembly under desertification and may contribute to the fragmentation of fungal association networks.

## Figures and Tables

**Table 1:** Methods used for the determination of soil physicochemical properties.

Parameter	Method	Standard
Alkali-hydrolyzable nitrogen	Alkali diffusion method	LY/T 1228-2015
Available phosphorus	Sodium bicarbonate extraction–molybdenum antimony colorimetric method	NY/T 1121.25-2012
Soil organic matter	External heating potassium dichromate oxidation method	NY/T 1211.6-2006
Total nitrogen	Kjeldahl method	NY/T 1121.24-2012
Total phosphorus	Alkali fusion–molybdenum antimony colorimetric method	NY/T 88-1988
Total potassium	Sodium hydroxide fusion method	NY/T 87-1988

NY/T, Agricultural Industry Standard of the People’s Republic of China; LY/T, Forestry Industry Standard of the People’s Republic of China.

## Data Availability

The ITS and AMF sequencing data generated in this study are publicly available in the NCBI Sequence Read Archive (SRA) under BioProject accession number PRJNA1455379 (https://www.ncbi.nlm.nih.gov/bioproject/PRJNA1455379, accessed on 20 April 2026).

## Supplementary Materials

The following supporting information can be downloaded at https://www.mdpi.com/article/10.3390/jof12060440/s1. Figure S1: Mantel analysis of the correlations between rhizosphere soil fungal α- and β-diversity and soil variables in *Stipa breviflora* (SB). Figure S2: Mantel analysis of the correlations between rhizosphere soil fungal α- and β-diversity and soil variables in *Artemisia frigida* (AF). Table S1: Effects of grassland type and plant species on fungal functional guilds. Figure S3: Correlation analysis of the relative abundance of fungal functional groups with AMF diversity. Figure S4: Spearman correlation analysis between relative abundances of fungal functional groups, AMF diversity, and fungal network topological indices. Figure S5: Spearman correlation analysis between soil factors and fungal network topological indices.
