# Supplementary material for: Desertification Drives Functional Reassembly of Rhizosphere Fungal Communities from Arbuscular Mycorrhizal Fungi to Dark Septate Endophytes in Temperate Grassland
Source: J Fungi (Basel). 2026 Jun 16;12(6):440. doi: 10.3390/jof12060440 (PMC13301376; doi:10.3390/jof12060440)
Supplement: Supplementary file 1 [file jof-12-00440-s001.zip › jof-4328915-supplementary.pdf]

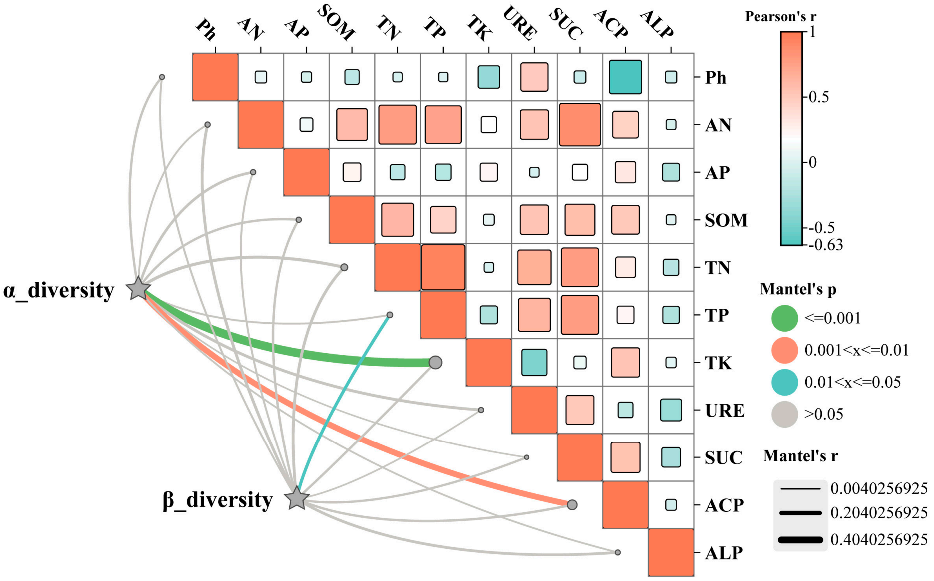

Figure S1. Mantel analysis of the correlations between rhizosphere soil fungal α- and β-diversity and soil variables in SB.

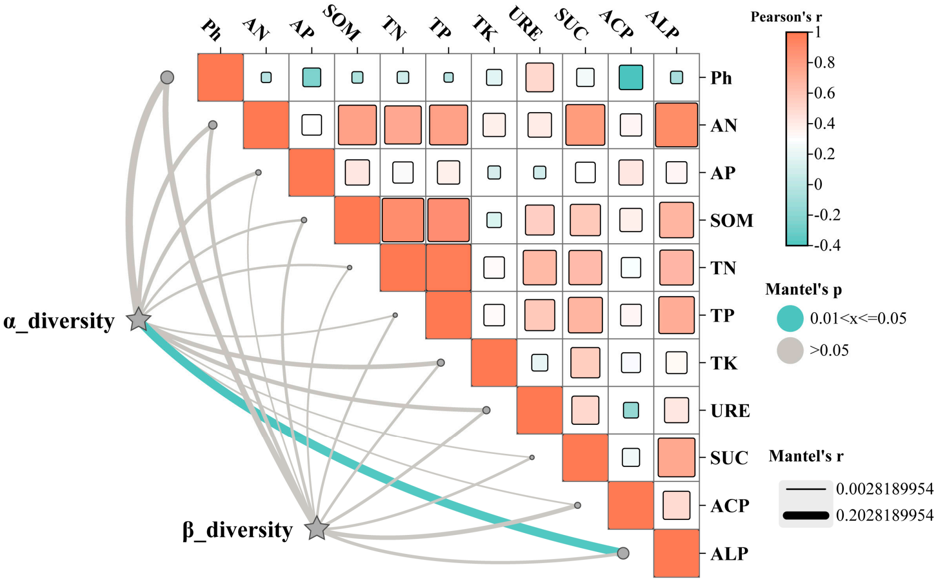

Figure S2. Mantel analysis of the correlations between rhizosphere soil fungal α- and β-diversity and soil variables in AF.

Table S1. Effects of grassland type and plant species on fungal functional guilds

	Plant	Grassland	Plant × Grassland
Plant Saprotroph	ns	*	ns
Plant Pathogen	ns	ns	ns
Endophyte	ns	***	ns

Animal Pathogen	ns	***	ns
Wood Saprotroph	ns	***	ns
Undefined Saprotroph	ns	***	ns
Fungal Parasite	ns	*	ns
Lichen Parasite	ns	*	ns
Dung Saprotroph	ns	ns	ns
Epiphyte	ns	ns	ns

Results of two-way ANOVA show the effects of desertification (Grassland), plant species (Plant), and their interaction (Grassland × Plant): ns, not significant; * $p < 0.05$; ** $p < 0.01$; *** $p < 0.001$.

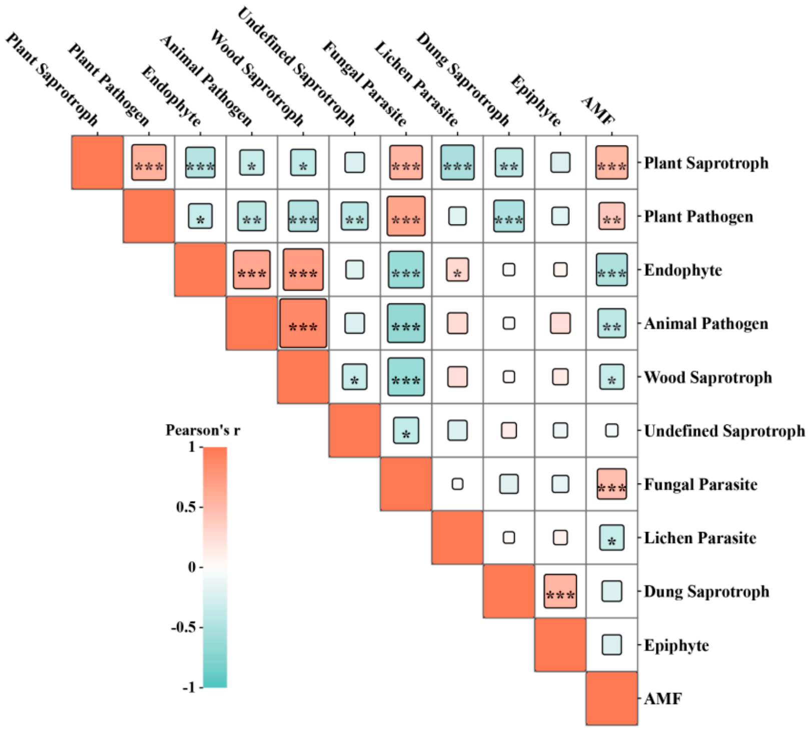

Figure S3. Correlation analysis of the relative abundance of fungal functional groups with AMF diversity, * $p < 0.05$; ** $p < 0.01$; *** $p < 0.001$.

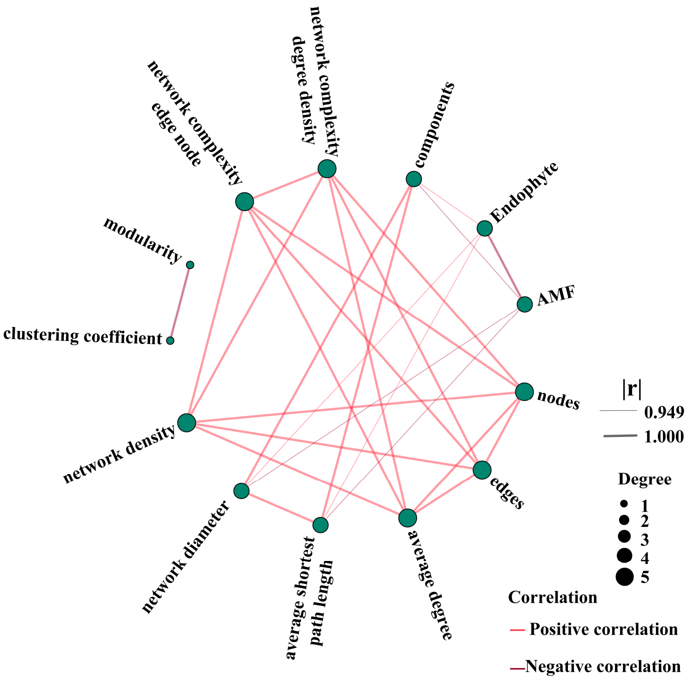

Figure S4. Spearman correlation network showing relationships among FUNGuild-inferred endophytic fungi, AMF diversity, and fungal network topological indices. Only significant correlations with |r| > 0.8 are displayed.

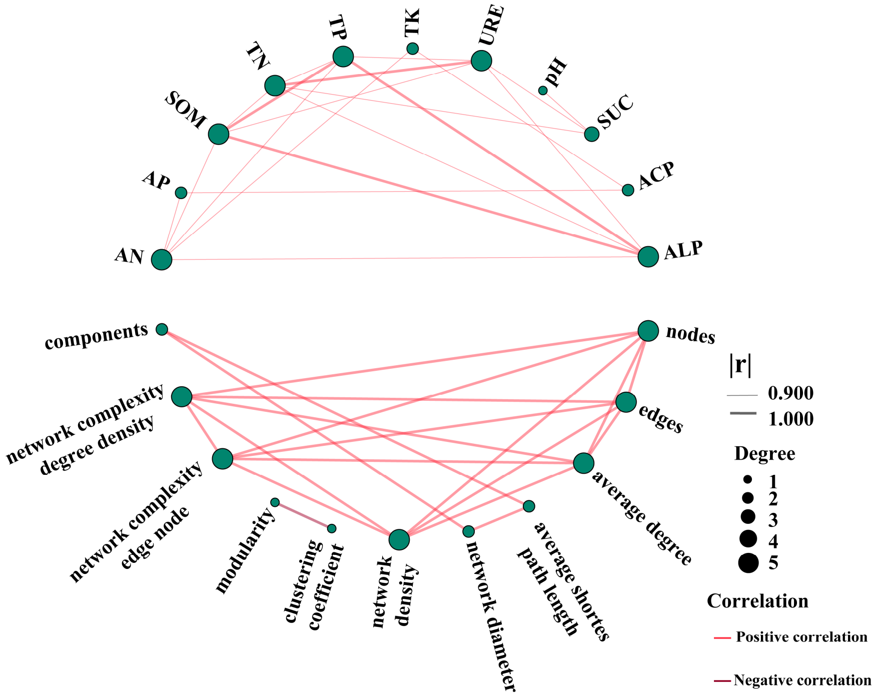

Figure S5. Spearman correlation network between soil factors and fungal network topological indices. Only significant correlations with |r| > 0.8 are shown.
